# Signaling Pathways Implicated in Carbon Nanotube-Induced Lung Inflammation

**DOI:** 10.3389/fimmu.2020.552613

**Published:** 2020-12-11

**Authors:** Jie Dong

**Affiliations:** Receptor Biology Laboratory, Toxicology and Molecular Biology Branch, Health Effects Laboratory Division, National Institute for Occupational Safety and Health, Centers for Disease Control and Prevention, Morgantown, WV, United States

**Keywords:** inflammation, type 2 immune response, carbon nanotube, immune cell, signaling pathway, transcription factor

## Abstract

Inflammation is a tissue response to a variety of harmful stimuli, such as pathogens, irritants, and injuries, and can eliminate insults and limit tissue damage. However, dysregulated inflammation is recognized as a cause of many human diseases, exemplified by organ fibrosis and cancer. In this regard, inflammation-promoted fibrosis is commonly observed in human lung diseases, such as idiopathic pulmonary fibrosis and pneumoconiosis. Carbon nanotubes (CNTs) are a type of nanomaterials with unique properties and various industrial and commercial applications. On the other hand, certain forms of CNTs are potent inducers of inflammation and fibrosis in animal lungs. Notably, acute inflammation is a remarkable phenotype elicited by CNTs in the lung during the early acute phase post-exposure; whereas a type 2 immune response is evidently activated and dominates during the late acute and chronic phases, leading to type 2 inflammation and lung fibrosis. Numerous studies demonstrate that these immune responses involve distinct immune cells, various pathologic factors, and specific functions and play crucial roles in the initiation and progression of inflammation and fibrosis in the lung exposed to CNTs. Thus, the mechanistic understanding of the immune responses activated by CNTs has drawn great attention in recent years. This article reviews the major findings on the cell signaling pathways that are activated in immune cells and exert functions in promoting immune responses in CNT-exposed lungs, which would provide new insights into the understanding of CNT-induced lung inflammation and inflammation-driven fibrosis, the application of CNT-induced lung inflammation and fibrosis as a new disease model, and the potential of targeting immune cells as a therapeutic strategy for relevant human lung diseases.

## Introduction

Carbon nanotubes (CNTs) are a category of cylindrical nanomaterials composed of either a single layer or concentric multiple layers of one-atom-thick carbon sheets, which are designated as single-walled carbon nanotubes (SWCNTs) and multi-walled carbon nanotubes (MWCNTs), respectively. In the past two decades, the increasing annual production of CNTs and CNT-containing materials and the expanding applications of CNTs in various industrial and commercial areas, such as electronics, energy, materials, and biomedical devices and drugs, have noticeably taken place owing to the unique properties of CNTs as new materials (1–3). However, some physicochemical properties of CNTs, such as the nano-scaled size, high aspect ratio, fiber-like shape, poor solubility, and substantial biopersistence, render CNTs to be respirable fibers, cause CNTs to act as foreign bodies after inhalation, and potentially link CNTs to toxic fibers with pathological activities, similar to asbestos. Indeed, a number of toxicological effects, exemplified by cytotoxicity, genotoxicity, and immunotoxicity, and pathological effects, exemplified by inflammation, fibrosis, and tumorigenesis, are markedly induced by certain types of CNTs in experimental animals, resulting in a serious concern over the potential adverse health effects of CNT exposure in human populations (4–9).

The most predominant CNT-induced pathological outcomes are recognized as the rapidly initiated and long-lasting inflammation and fibrosis in the lung in exposed experimental animals. Accumulating phenotypic observations exhibit that CNT-triggered lung inflammation and fibrosis share high similarities with those occurring in a variety of human lung diseases, such as idiopathic pulmonary fibrosis, silicosis, and asbestosis, regarding pathologic features. Moreover, mechanistic analyses reveal that the systemic, cellular, and molecular activities during the development of inflammation and fibrosis in CNT-exposed lungs are in agreement with the overall understanding of these pathologic processes derived from relevant human diseases and animal disease models, indicating the potential of CNT-exposed animals to serve as a new disease model. These findings markedly escalate the interest and significance of elucidating inflammation and fibrosis induced by CNT exposure in the lung. It is noticeable that similar to the scenarios observed in many chronic diseases, exemplified by idiopathic pulmonary fibrosis, liver fibrosis, and systemic sclerosis, inflammation and fibrosis in CNT-exposed lungs are demonstrated to mechanistically interact with each other through the activities of effector cells, soluble mediators, and ECM (10–14). In this regard, the time- and context-dependent activation, mode of action, and function of immune responses play critical roles in regulating the initiation and progression of inflammation and fibrosis in the lung during CNT exposure. Therefore, elucidating CNT-induced immune responses is a requisite step for understanding lung pathology triggered by CNTs.

The inducible regulation of intracellular signaling plays a central role in physiological conditions and meanwhile functions as the key means to respond to endogenous and exogenous stresses in multicellular organisms. Dysregulation of cell signaling is responsible for the deleterious effects that lead to disease initiation and development. Accordingly, following the phenotypic observation of CNT-induced inflammation and fibrosis, in recent years great efforts have been made into the identification of crucial cell signaling mechanisms that are induced by CNT exposure and implicated in immune response activation and function in the lung. To dissect the cellular and molecular mechanisms underlying CNT-induced inflammation, in this article, recent advances into the framework encompassing key signaling elements in immune cells in CNT-exposed lungs are discussed. Analysis in this aspect is anticipated to enhance the understanding of lung pathology induced by CNTs and meanwhile provide mechanistic basis for the development of CNT-exposed animals as a new disease model for lung inflammation and fibrosis.

## CNT-Induced Pulmonary Inflammation and Fibrosis

**Figure 1:**
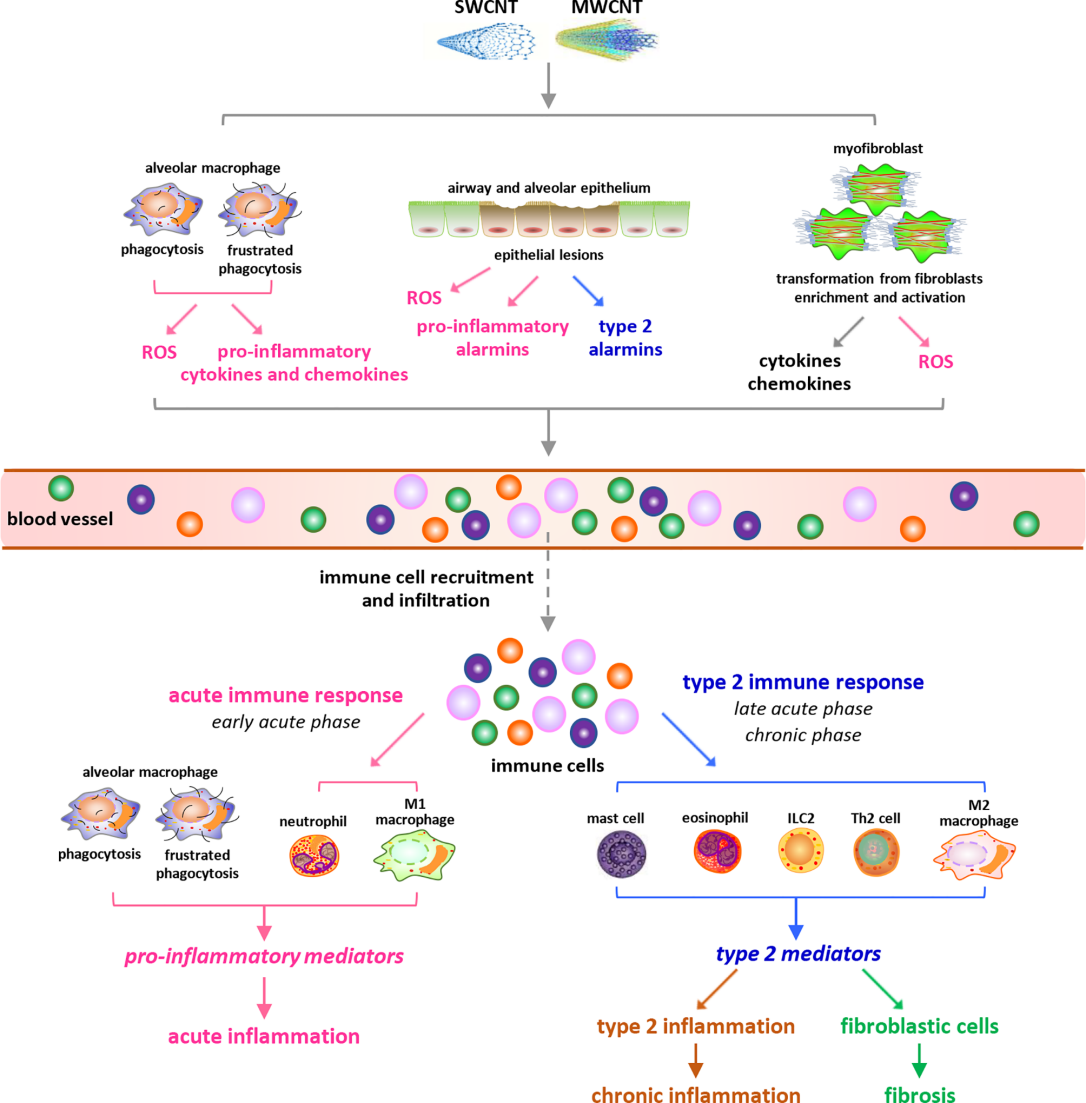
Overview of carbon nanotube (CNT)-induced lung inflammation. Under exposure to CNTs, various immune cells are recruited from blood vessels and infiltrate into lung tissues, triggered by cytokines, chemokines, ROS, and alarmins that are induced by CNTs through distinct mechanisms. During the early acute phase response, neutrophils and M1 macrophages are dominant and active to produce pro-inflammatory cytokines, chemokines, and growth factors, resulting in acute inflammation. Whereas during the late acute phase response, the immune cells implicated in type 2 immune response are dominant and produce type 2 cytokines and mediators, leading to type 2 inflammation. The activation of type 2 immune response mediates the transition from acute inflammation to chronic inflammation and promotes the development of lung fibrosis.

Inflammation and fibrosis in the lung are the most predominant pathological phenotypes in CNT-exposed experimental animals, concluded from numerous studies carried out in the past decade. In keeping with the accumulative findings achieved in this area, a number of recent review articles have summarized the progress in different aspects of CNT-induced lung inflammation and fibrosis (10, 11, 14–17). To avoid repetition, here the current knowledge on this topic is introduced concisely and comprehensively as background information (**Figure 1**). For more detailed and profound understanding, the previously published review papers, as well as the research papers cited therein, are recommended to refer to.

Upon exposure to CNTs through inhalation, intratracheal instillation, or pharyngeal aspiration, acute inflammation is rapidly induced in the lung, marked by pronounced recruitment and infiltration of inflammatory cells, such as neutrophils, macrophages, and lymphocytes, and copious production and secretion of pro-inflammatory and pro-fibrotic cytokines, chemokines, and growth factors, such as TNF-α, IL-1β, IL-6, MCP-1, TGF-β1, and PDGF. Accompanying the acute inflammation, CNTs trigger a rapid-onset fibrotic response, indicated by increased deposition of ECM in alveolar septa starting as early as day 1 post-exposure (18, 19). The acute inflammatory and fibrotic responses are maintained at high levels within 7 days post-exposure. During the early phase of this stage, the infiltration and activation of neutrophils and traditionally activated M1 macrophages are dominant, resulting in acute inflammation; whereas during the late phase, type 2 immune response becomes overwhelming in the lung. Type 2 immune response is activated through the induction and functionalization of Th2 lymphocytes and alternatively activated M2 macrophages, which produce type 2 cytokines and mediators that function in suppressing acute inflammation and promoting Th2-driven type 2 inflammation and organ fibrosis. The fibrotic response is induced through the enriched and activated fibroblasts and myofibroblasts, which are fibrosis effector cells responsible for producing ECM proteins (10, 14). Thereafter, acute inflammatory and fibrotic responses decline, but the chronic consequences develop (18, 20). The chronic phenotypes are fully established by day 28 and persist for at least 1 year post-exposure, which are characterized with interstitial fibrosis, chronic inflammation, and granulomas. CNT-induced lung fibrosis is featured with thickened alveolar septa, formation of fibrotic foci and epithelioid granulomas, enrichment and activation of fibroblasts and myofibroblasts, elevated expression of fibrosis marker proteins, excessive deposition of ECM, and lack of massive alveolar epithelial cell death (15). The chronic inflammation is known to implicate M2 macrophages, T lymphocytes, and the increased levels of certain pro-inflammatory and pro-fibrotic cytokines, chemokines, and growth factors; nevertheless, it awaits further analysis for its features, activities, and functions in CNT-exposed lungs (14). In aggregate, these observations demonstrate that immune system activation controls the initiation, progression, and maintenance of CNT-triggered lung inflammation, and meanwhile plays critical roles in promoting CNT-induced lung fibrosis through producing a variety of pro-fibrotic mediators. Thus, a comprehensive mechanistic understanding of CNT-activated immune responses would provide new insights into CNT lung pathology, as well as enhance the overall understanding of human lung diseases involving inflammation and fibrosis.

Importantly, a few recent studies demonstrate that certain pro-inflammatory and pro-fibrotic responses identified in experimental animals are induced in CNT-exposed workers as well. For instance, the levels of TNF-α, IL-1β, IL-4, IL-5, IL-6, and IL-8 in sputum, and TNF-α, IL-1β, and IL-4 in serum, of the workers occupationally exposed to MWCNTs are significantly higher than controls (21); the levels of CCL20, sIL-1RII, and FGF-BASIC are elevated in the serum of MWCNT-exposed workers, compared with controls (22); and the level of ICAM-1 in the blood shows a dose-dependent upward trend in MWCNT-exposed workers, compared with unexposed group, at two time points examined (23). Although the investigation on CNT-induced effects in humans is at an early stage, these findings indicate that inflammation and fibrosis are potential pathological outcomes of CNT exposure in human populations.

Mechanistic studies reveal that CNT exposure may trigger the infiltration of immune cells, a hallmark and initial step of inflammation, in the lung through multiple ways (**Figure 1**). First, CNTs cause phagocytosis and frustrated phagocytosis in alveolar macrophages, resulting in elevated production of ROS that induces inflammation and tissue damage (5, 9, 24, 25). SWCNTs induce a dose-dependent accumulation of 4-HNE (a marker of lipid peroxidation) and a time- and dose-dependent depletion of GSH (a major antioxidant) as early as 1 day post-exposure in mouse lungs, indicating oxidative stress as a rapid response to SWCNT exposure (26). MWCNTs (XNRI MWNT-7) markedly increase ROS production in alveolar macrophages, as well as the levels of oxidative stress markers 8-OHdG, γH2AX, and 4-HNE, in mouse lungs; and these increases are evidently more striking in Nrf2 KO lungs than in WT lungs. Correspondingly, the numbers of immune cells, including granulocytes, macrophages (Mac2+), T cells (CD3+), and B cells (B220+), are noticeably higher in Nrf2 KO lungs than in WT lungs (27). These findings indicate the critical role of ROS in stimulating immune cell infiltration and initiating immune responses in the lung exposed to CNTs. Second, alveolar macrophages can be activated by CNTs to produce pro-inflammatory cytokines and chemokines, which trigger the recruitment and infiltration of inflammatory cells, such as neutrophils and monocytes. Alveolar macrophage depletion with liposomal clodronate impairs the induction of TNF and IL-6 by MWCNTs 12 h post-exposure and attenuates the influx of neutrophils induced by MWCNTs 24 h post-exposure in mouse lungs; and adoptive transfer of alveolar macrophages into alveolar macrophage-depleted mice partially rescues the induction of TNF and, conditionally, IL-6, and the infiltration of neutrophils by MWCNTs in the lung (28). Thus, the production of pro-inflammatory mediators by alveolar macrophages in CNT-exposed lungs may trigger the onset of inflammation. Third, CNTs can penetrate airway and alveolar epithelium to induce epithelial lesions, which result in increased production and secretion of alarmins by epithelial cells, in the lung (14). Among CNT-induced alarmins, HMGB1 promotes acute inflammation by increasing IL-1β secretion, whereas IL-25, IL-33, and TSLP may trigger the recruitment of type 2 immune cells leading to the activation of type 2 immune response, in CNT-exposed lungs. The induction and roles of HMGB1 and IL-33 in CNT-exposed lungs are discussed in detail in later sections. Fourth, myofibroblasts are highly enriched during fibrotic response to CNT exposure in the lung. Myofibroblasts exhibit high levels of constitutive and induced production and secretion of cytokines, chemokines, and ROS, which may contribute to the infiltration of immune cells as well (15). Combined, these effects of CNTs enable the recruitment of different types of immune cells and the activation of distinct immune responses in CNT-exposed lungs.

The immune responses induced by CNTs lead to the elevated production of pro-inflammatory and pro-fibrotic soluble factors, such as cytokines and growth factors, which activate certain cell signaling pathways in fibroblastic cells and thereby promote CNT-induced lung fibrosis. For instance, pro-inflammatory cytokines, such as TNF-α and IL-1β, can activate the canonical NF-κB signaling to upregulate the expression of pro-fibrotic mediators TIMP1 and OPN in fibroblasts and myofibroblasts; type 2 mediators OPN and TGF-β1 can activate the canonical, Smad-dependent TGF-β signaling to induce the expression of fibrotic proteins, such as α-SMA, Collagen I, and fibronectin, in fibroblasts and myofibroblasts; and type 2 mediator TIMP1 can activate ERK signaling in fibroblasts to promote fibroblast proliferation, in CNT-exposed mouse lungs (14). Thus, immune responses generate microenvironmental cues that promote the initiation and progression of lung fibrosis induced by CNTs.

The immune responses manipulate the rapid-onset of acute inflammation, propagation of pro-inflammatory cues, recruitment, differentiation, and polarization of immune cells of different functions, transition from a pro-inflammatory immune response to a type 2 immune response, and progression from acute inflammation to chronic inflammation. These functions are exerted and controlled by the induction and activation of cell signaling pathways and their target functional proteins, molecular mediators, and cellular processes. As such, the time- and context-dependent signaling pathways activated in immune cells play central roles in the orchestration of immune responses in CNT-exposed lungs. In this respect, a number of important pathways have been identified to underlie CNT-induced immune responses and pathological outcomes in the lung, which are specifically discussed in this article.

## Cell Signaling Associated with CNT-Induced Acute Inflammation in the Lung

**Figure 2:**
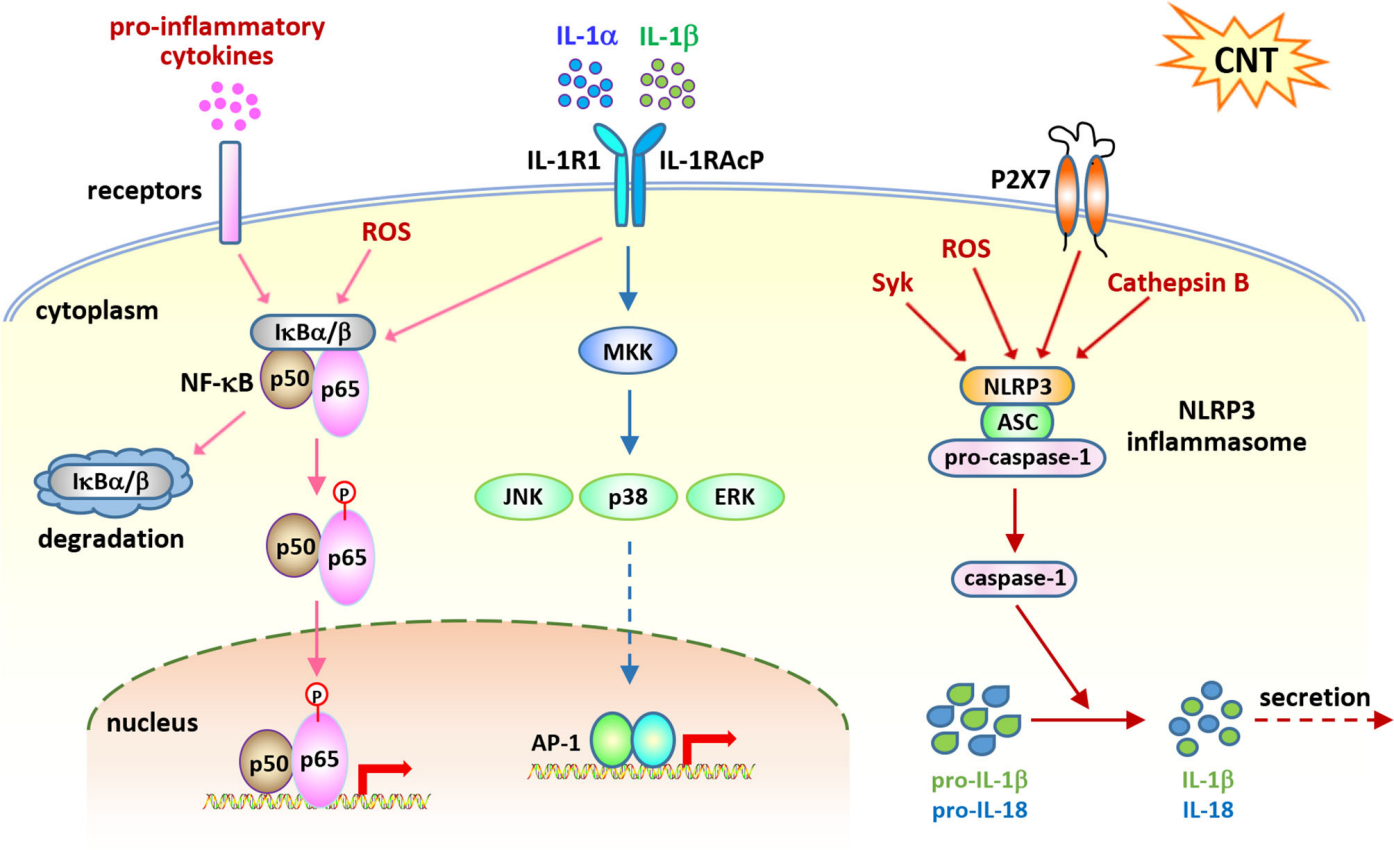
Cell signaling pathways activated in acute inflammation induced by carbon nanotubes (CNTs). NF-κB signaling is activated by CNTs and upregulates the expression of a variety of pro-inflammatory cytokines, such as TNF-α, IL-1α, and IL-1β, which in turn activate this signaling pathway. IL-1R signaling is shown to play a critical role in promoting CNT-induced acute inflammation in the lung, which is activated by IL-1α and IL-1β. NLRP3 inflammasome signaling is activated by CNTs and generates mature IL-1β and IL-18 for secretion and function. These three pathways represent the molecular basis for CNT-triggered acute inflammation.

Acute inflammation is an immediate defense to a diversity of environmental insults, such as microbial infections and foreign body deposition. Neutrophils and macrophages are the key frontline players in this response with multiple protective functions, such as killing pathogens and engulfing and clearing foreign bodies. In CNT-exposed lungs, acute inflammation is rapidly induced within 1 day post-exposure and exhibits as a dominant phenotype in early acute phase response, marked by the increased numbers of neutrophils and macrophages and elevated levels of pro-inflammatory cytokines and chemokines (14, 18, 26). A few critical pathways have been investigated to elucidate the mechanisms underlying the striking acute inflammatory response, which disclose the early activities that initiate CNT-induced pathologic effects (**Figure 2**). The functional studies performed in knockout mice are listed in **Table 1**.

**Table 1:** Functional studies of signaling molecules in carbon nanotube (CNT)-induced pulmonary immune responses using knockout (KO) mice.

KO mice	CNTs	Effects	Findings in KO mice	References
IL-1R KO	long, rod-like MWCNTs (XNRI MWNT-7) OD: >50 nm L: ~13 µm long, tangled MWCNTs OD: 8–15 nm L: 10–50 µm	acute inflammation	Long, rod-like MWCNTs, but not long, tangled MWCNTs, increase neutrophils in BAL and pro-inflammatory cytokine and chemokine expression in lung tissues 16 h post-exposure in WT mice, which are attenuated in IL-1R KO mice. Long, rod-like MWCNTs induce Th2-type inflammation on day 28 post-exposure in WT mice, which is not affected in IL-1R KO mice.	(29)
IL-1R KO	MWCNTs (XNRI MWNT-7) D: 49±13.4 nm L: 3.86±1.94 µm	acute inflammation	MWCNT-induced acute inflammation is suppressed in IL-1R KO mice 24 h post-exposure, compared with WT mice, whereas IL-1R KO does not suppress inflammation on day 28 post-exposure, determined by the numbers of total cells, mononuclear cells, and neutrophils and the levels of IL-6, IL-12p40, and CXCL1 in BAL.	(30)
caspase-1 KO	MWCNTs (FA-21) D: 27 nm L: 5–15 µm	acute inflammation	The level of IL-1β, the number of total cells, and the number of neutrophils in BAL are significantly increased by MWCNTs on day 1 post-exposure in WT mice, but not in caspase-1 KO mice. The number of eosinophils in BAL is significantly increased by MWCNTs on day 1 post-exposure in WT mice, which is not affected in caspase-1 KO mice.	(31)
ST2 KO	MWCNTs D: 22.5±1.3 nm L: 10–100 µm	chronic inflammation type 2 immune response	The induction of inflammation, fibrosis, and functional damage by MWCNTs in the lung on day 30 post-exposure is inhibited in mast cell-deficient *Kit^W-sh^* mice and ST2 KO mice, compared with WT mice. Reconstitution of *Kit^W-sh^* mice with WT BMMCs restores these MWCNT-induced pathological outcomes, whereas that with ST2 KO BMMCs does not.	(32)
IL-13 KO	MWCNTs (FA-21) D: 27 nm L: 5–15 µm	type 2 immune response	MWCNTs increase eosinophils in WLL 24 h post-exposure in WT mice, but not in IL-13 KO mice.	(33)
IL-33 KO	MWCNTs (FA-21) D: 27 nm L: 5–15 µm	type 2 immune response	MWCNTs increase eosinophils in WLL 24 h post-exposure in WT mice, but not in IL-33 KO mice.	(33)
IL-33 KO	MWCNTs D: 22.5±1.3 nm L: 10–100 µm	chronic inflammation type 2 immune response	The number of eosinophils in BAL is increased by MWCNTs on day 30 post-exposure in WT mice, which is attenuated in IL-33 KO mice. MWCNTs induce fibrosis near the airways in WT mice, but not in IL-33 KO mice, on day 30 post-exposure.	(34)
STAT6 KO	MWCNTs (XNRI MWNT-7) D: 49±13.4 nm L: 3.86±1.94 µm	type 2 immune response	The level of IL-5 is elevated by MWCNTs in the BAL from WT mice, but not in the BAL from STAT6 KO mice, 24 h post-exposure. MWCNT-induced fibrotic phenotype is weakened in STAT6 KO lung tissues, compared with WT lung tissues, on day 28 post-exposure.	(30)

D, diameter; L, length; OD, outside diameter; WLL, whole lung lavage.

## NF-κB Signaling 

The transcription factor NF-κB plays critical roles in immune responses through upregulating the transcription of a wide range of genes. A number of NF-κB target genes encode the proteins that function as inducers, mediators, and effectors in activating inflammatory networks upon exposure to stimuli. Excessive and prolonged NF-κB activation is implicated in a long list of inflammatory diseases, such as asthma, rheumatoid arthritis, inflammatory bowel disease, and multiple sclerosis (35–41). As such, activation of NF-κB signaling is regarded as one of the most predominant events that control inflammatory responses in disease or under stress.

Studies performed in various types of cultured cells, such as epithelial cells, endothelial cells, and mesothelial cells, reveal that SWCNTs and MWCNTs are capable of inducing NF-κB activation under different conditions (42–47). Importantly, SWCNTs and MWCNTs activate NF-κB pathway and elevate the expression of NF-κB-target genes that encode pro-inflammatory cytokines and chemokines, such as TNF-α, IL-1β, IL-6, and MCP-1, in mouse RAW264.7 macrophages (48, 49). These *in vitro* studies suggest the potential involvement of NF-κB in CNT-induced pathologic effects in the lung, including the onset of inflammation. Pathway analysis of Affymetrix microarray data reveals that NF-κB-associated inflammatory responses and downstream signals regulating tissue remodeling are important factors for the pathologic outcomes induced by SWCNTs in mouse lungs (50, 51). Meanwhile, upstream regulator and network analysis of Illumina microarray data identifies NF-κB signaling as one of the major networks that are activated by MWCNTs in mouse lungs (52). These genome-wide gene expression studies provide the evidence for the overall activation of NF-κB by SWCNTs and MWCNTs in the lung.

**Figure 3:**
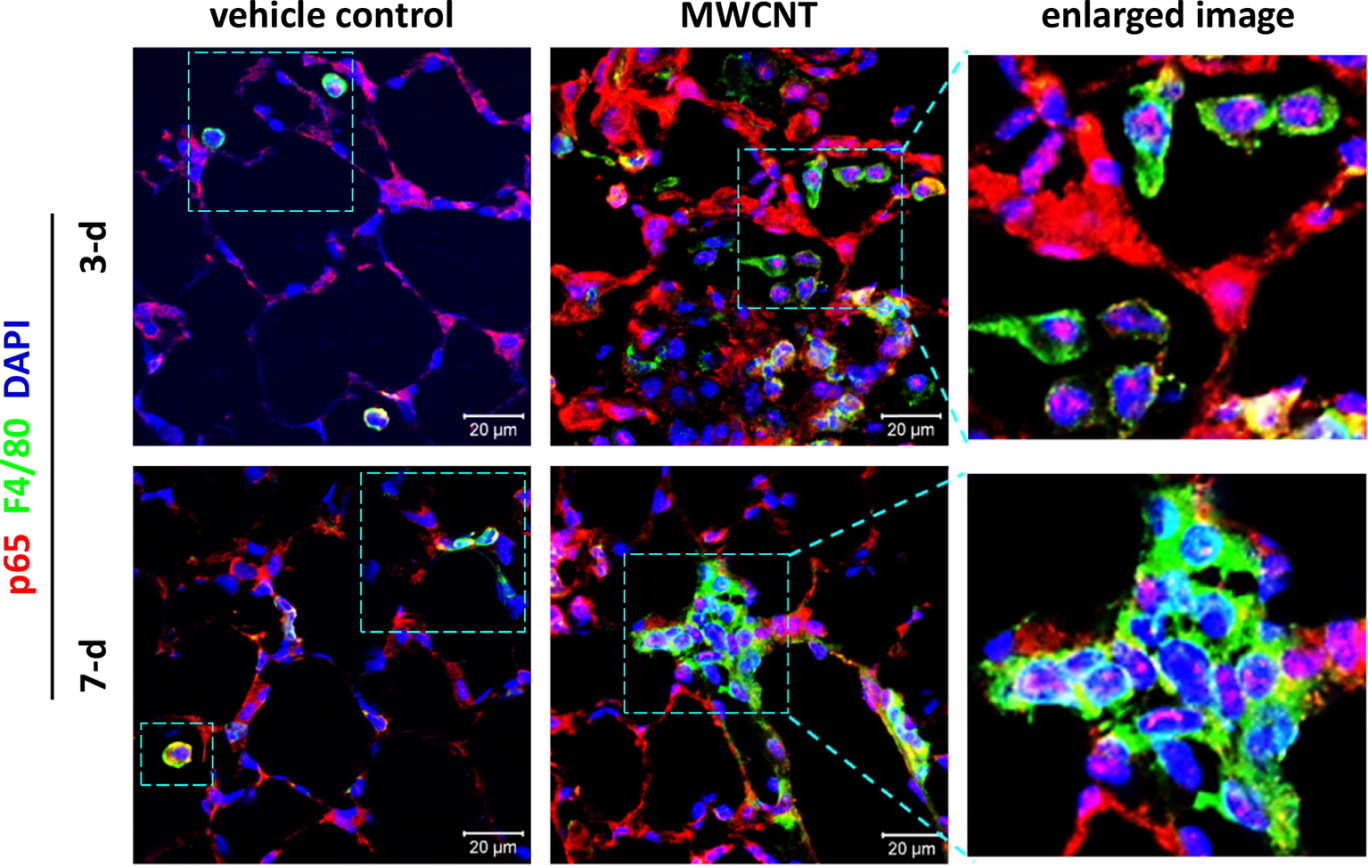
Activation of NF-κB in macrophages during acute inflammation in the lung of multi-walled carbon nanotube (MWCNT)-exposed C57BL/6J mice. Nuclear p65 in macrophages is examined by double immunofluorescence staining of p65 (red) and F4/80 (green), with DAPI nuclear staining (blue), following the method described previously (53). Pink color generated from the overlap of red and blue indicates nuclear p65, which demonstrates the activation of NF-κB. During both the early acute phase and late acute phase, NF-κB is remarkably activated by MWCNTs in macrophages in mouse lungs.

To determine the effect of CNTs on NF-κB signaling in macrophages during acute inflammation in mouse lungs, nuclear translocation of NF-κB subunit p65, a marker for NF-κB activation, was examined by double immunofluorescence staining of p65 (red) and the macrophage marker F4/80 (green), with DAPI nuclear staining (blue), on lung tissue sections of WT C57BL/6J mice. The effect of inflammagenic MWCNTs (XNRI MWNT-7; outside diameter: 49±13.4 nm; length: 3.86±1.94 µm) was studied on days 3 and 7 post-exposure, which represent the early acute phase and late acute phase of exposure, respectively. It is demonstrated that NF-κB is markedly activated in macrophages by MWCNTs during the entire acute phase response in the lung, indicated by nuclear NF-κB with pink color generated from the overlap of the red color (p65 staining) and the blue color (nuclear staining) in images (**Figure 3**). This finding provides direct *in vivo* evidence for the activation of NF-κB in macrophages in MWCNT-exposed lungs, and also reveals the mechanistic basis for the elevated expression and secretion of NF-κB-regulated pro-inflammatory cytokines and chemokines in the lung exposed to CNTs.

## IL-1α/β–IL-1R Signaling

IL-1α and IL-1β are potent pro-inflammatory cytokines with elevated levels in various inflammatory diseases. In addition, they are induced and secreted, mainly by monocytes and macrophages, as an acute immune response to infection, lesion, and stress. They bind to IL-1R, activate the NF-κB and MAPK signaling pathways, and induce the expression of the genes encoding pro-inflammatory cytokines and chemokines, thereby exerting pro-inflammatory functions (54–59). Thus, together with the TNF-α−TNFR pathway and the IL-6−IL-6R pathway, the IL-1α/β–IL-1R signaling is recognized as a major player in the initiation and maintenance of inflammation.

The IL-1α/β-activated, IL-1R-mediated signaling has drawn attention when studying CNT-induced inflammation, owing to the markedly increased levels of IL-1α and IL-1β in CNT-exposed lungs observed in numerous animal studies. The level of IL-1α in BAL obtained from mice exposed to MWCNTs (XNRI MWNT-7) is significantly elevated on days 1, 3, 7, and 14 post-exposure (18, 30). The level of IL-1β in BAL is significantly increased 40 h, and on days 1, 3, 7, and 28, post-exposure to SWCNTs in mice (26, 60), and on days 1, 3, and 21 post-exposure to MWCNTs in mice (18, 30, 61–63).

In this scenario, the role of IL-1α/β in CNT-induced lung inflammation has been investigated, with the facilitation of IL-1R KO mice and IL-1R antagonists (29). Long, rod-like MWCNTs (outside diameter: >50 nm; length: ~13 µm), but not long, tangled MWCNTs (outside diameter: 8-15 nm; length: 10-50 µm), induce strong pulmonary neutrophilia, demonstrated by increased number of neutrophils in BAL, as well as the expression of pro-inflammatory cytokines and chemokines, such as TNF-α, IL-1β, CXCL1, CXCL2, and CXCL5, in lung tissues, 16 h post-exposure in WT mice. These phenotypes are significantly attenuated in WT mice that are pre-treated with IL-1R antagonists, etanercept and/or anakinra, to block IL-1α/β–IL-1R signaling and in IL-1R KO mice. In contrast, deficiency of IL-1R does not affect long, rod-like MWCNT-induced Th2-type inflammation, indicated by IL-13 expression and mucus production, on day 28 post-exposure. Similarly, in another study, it is demonstrated that XNRI MWNT-7 MWCNT-induced acute inflammation is suppressed in IL-1R KO mice on day 1 post-exposure, compared with WT mice, whereas IL-1R deficiency does not suppress inflammation on day 28 post-exposure, determined by the numbers of total cells, mononuclear cells, and neutrophils and the levels of IL-6, IL-12p40, and CXCL1 in BAL (30). These studies therefore highlight the critical role of IL-1α/β–IL-1R signaling in the onset of acute inflammatory response induced by MWCNT exposure in the lung. Furthermore, the reduced induction of NF-κB-regulated pro-inflammatory cytokines and chemokines by impaired IL-1α/β–IL-1R signaling observed in these studies indicates NF-κB as a downstream target that is activated by IL-1α/β–IL-1R signaling in CNT-exposed lungs. MWCNTs have been shown to increase phosphorylation of ERK1/2 in mouse RAW264.7 macrophages, which is crucial to the induced expression of COX-2 by MWCNTs (64). However, whether the MAPK pathway is activated through IL-1α/β–IL-1R signaling and contributes to inflammation in CNT-exposed lungs awaits to be investigated.

## NLRP3 Inflammasome Activation

Inflammasomes function as key components of cytosolic sensors in detecting intracellular and extracellular signals and initiating innate immune responses to protect from microbe infection and tissue injury (65–68). They are large multiple protein complexes with a few types identified. Each type of inflammasome has a distinct protein composition and is activated by distinct and specific stimuli, such as microbial pathogens and stressors. NLRP3 inflammasome is the most well-studied complex and can be activated by a variety of pathogens, endogenous danger signals, and environmental stimuli. NLRP3 inflammasome is composed of the NLR protein NLRP3, the adaptor protein ASC, and the effector proteolytic enzyme caspase-1 (pro-caspase-1). Upon exposure to stimuli, NLRP3 protein is activated and oligomerized, and recruits ASC and pro-caspase-1, resulting in the formation of NLRP3 inflammasome, which then leads to the cleavage of pro-caspase-1 to active caspase-1. The active caspase-1 in turn cleaves inert pro-IL-1β and pro-IL-18 to generate mature, active IL-1β and IL-18, which are then secreted from the cell and play pro-inflammatory functions. As such, the activation of NLRP3 inflammasome is required for the production of IL-1β and IL-18 during inflammatory response.

The increased level of IL-1β in BAL induced by SWCNTs and MWCNTs in mice prompted the investigation of the activation of NLRP3 inflammasome by CNTs. A number of studies reveal that SWCNTs and MWCNTs increase NLRP3 inflammasome-mediated secretion of IL-1β and IL-18 in mouse and human macrophages *in vitro* and *ex vivo* (62, 69–74). For instance, long, needle-like, but not long, tangled, MWCNTs increase the secretion of IL-1β from LPS-primed human monocyte-derived macrophages, examined by ELISA, as well as the level of cleaved, active IL-1β, determined by immunoblotting. The induction of secreted IL-1β by MWCNTs is markedly attenuated by NLRP3 siRNA, compared with control siRNA, and by P2X7 inhibitor or siRNA, ROS inhibitor, cathepsin B inhibitor, and Syk inhibitor, which are known to impair the activation of NLRP3 inflammasome (73). Importantly, the level of IL-1β in BAL is increased by MWCNTs on day 1 post-exposure in WT mice, which is abolished in caspase-1 KO mice. Coincidently, the number of neutrophils, but not eosinophils, in BAL is significantly reduced in caspase-1 KO mice, compared with WT mice, on day 1 post-exposure to MWCNTs. This study therefore provides *in vivo* evidence for the activation of NLRP3 inflammasome and its critical role in increasing IL-1β secretion and inducing acute inflammation in MWCNT-exposed lungs (31). A recent study demonstrates that the level of IL-18 in BAL collected from MWCNT-exposed mice on day 1 post-exposure is also significantly elevated, which supports the activation of NLRP3 inflammasome by MWCNTs in the lung (75). Together, these findings identify that NLRP3 inflammasome is a target signaling of CNTs in lung cells, such as monocyte-derived macrophages and alveolar macrophages, and promotes the acute inflammatory response elicited by CNTs in the lung.

The pro-inflammatory alarmin HMGB1 exhibits an elevated level in BAL fluid obtained from mice exposed to SWCNTs or MWCNTs (31, 76). It is shown that the expression and secretion of HMGB1 are induced in C10 mouse epithelial cells by MWCNTs *in vitro*, suggesting epithelial cells are a source of HMGB1 in the lung when exposed to MWCNTs (31). Importantly, facilitated with anti-HMGB1 neutralizing antibodies and caspase-1 KO mice, it is revealed that HMGB1 increases IL-1β secretion by activating NLRP3 inflammasome and thereby promotes acute inflammation in mouse lungs exposed to MWCNTs (31). These studies thus demonstrate an alarmin-involved mechanism for MWCNT-induced acute inflammation in the lung.

## Cell Signaling Implicated in CNT-Induced Type 2 Inflammation in the Lung

**Figure 4:**
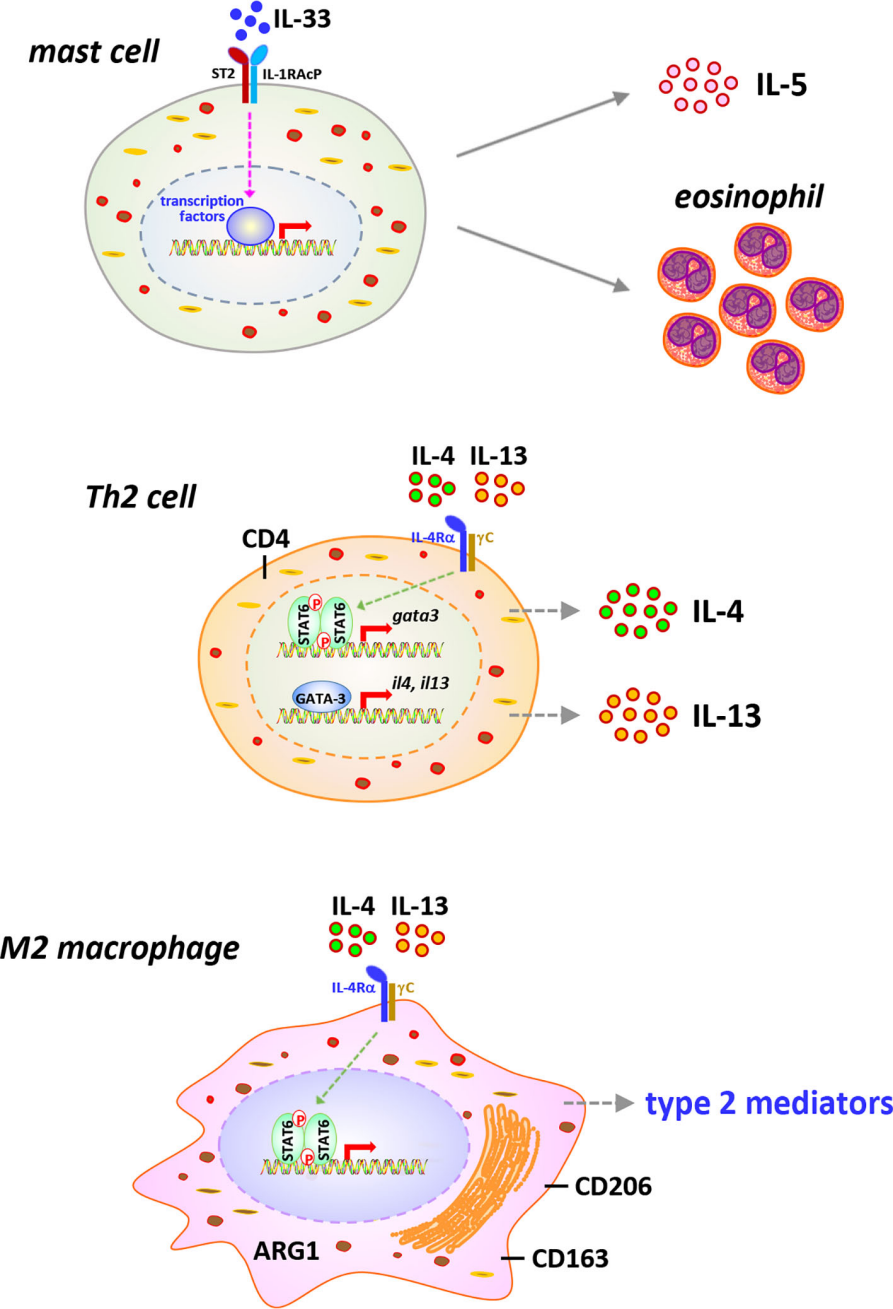
Immune cells and signaling pathways implicated in type 2 immune response in carbon nanotube (CNT)-exposed lungs. Type 2 alarmin IL-33 is induced and activates mast cells, leading to increased secretion of type 2 cytokine IL-5 and recruitment of eosinophils, in CNT-exposed lungs. CNTs induce the differentiation of Th2 cells and the activation of STAT6 and GATA-3 in Th2 cells, resulting in the expression and secretion of type 2 cytokines IL-4 and IL-13, in the lung. Furthermore, the polarization of M2 macrophages is induced by CNTs in the lung. M2 macrophages exhibit STAT6 activation and produce various type 2 mediators, which function in suppressing acute inflammation, developing type 2 inflammation and chronic inflammation, and promoting lung fibrosis.

Type 2 inflammation is characterized by the activation of type 2 immune cells, such as eosinophils, mast cells, ILC2s, Th2 lymphocytes, and M2 macrophages, and the production of type 2 cytokines and mediators, which exert functions in suppressing acute inflammation and promoting chronic inflammation, wound healing, and organ fibrosis (12, 77–81). It has been revealed that during the late acute phase and chronic phase responses to CNT exposure, type 2 inflammation presents as the dominant immune phenotype and promotes the development of fibrosis and chronic inflammation in the lung, although certain type 2 immune cells, such as eosinophils, Th2 cells, and M2 macrophages, and type 2 factors, such as IL-33 and IL-5, are observed to be induced by CNTs at mild levels during early acute phase as well (10, 30, 31, 33, 52, 82). In this regard, the cell signaling pathways that are activated during type 2 immune response and contribute to pathologic outcomes have been studied in CNT-exposed lungs in a few aspects (**Figure 4**). The functional analyses carried out using knockout mouse models are listed in **Table 1**.

## IL-33−ST2 Signaling

IL-33 is a cytokine of the IL-1 family and functions as an alarmin that activates immune responses under infection, injury, environmental stress, and a variety of diseases, such as asthma and COPD. It has drawn great attention because of its essential role in evoking type 2 immune response through activating the early effector cells in type 2 inflammation, such as mast cells, eosinophils, and ILC2s. IL-33 acts as a cytokine, *via* binding to its specific receptor ST2 and recruiting the co-receptor IL-1RAcP, to induce the activation of NF-κB and MAPK (JNK, ERK, and p38) signaling. One of the major outcomes from the activation of this IL-33−ST2 signaling is the production of type 2 cytokines, such as IL-4, IL-5, and IL-13, which provides the original source of these cytokines for eliciting the key events in type 2 immune response, more specifically, the differentiation and activation of Th2 cells and M2 macrophages (83, 84).

Consistent with the investigation on the activation of type 2 immune response in CNT-exposed lungs, the upstream IL-33−ST2 signaling has been observed in several studies. The levels of IL-33 in BAL and lung tissues are significantly elevated by MWCNTs in mice during both the acute and chronic responses, indicating the possible role of IL-33 in CNT-activated lung pathology (32, 33, 85, 86). Epithelial cells can be injured by a diversity of insults and then produce and secrete alarmins, including IL-33, and therefore are regarded as a major source for induced IL-33 production (12, 78, 87). Indeed, in MWCNT-exposed mouse lungs, IL-33+ type II pneumocytes (surface epithelial cells of the alveoli) are present in the vicinity of alveolar macrophages phagocytosing MWCNTs or free MWCNTs, but not in the areas lacking MWCNTs, demonstrating MWCNT-stimulated production of IL-33 by epithelial cells (33). Importantly, the implication of IL-33−ST2 signaling in CNT-induced pathological effects in the lung has been examined by using genetically engineered mice and blocking antibodies. First, MWCNTs induce lung inflammation, lung fibrosis, and impaired lung functions on day 30 post-exposure in WT mice, which are remarkably attenuated in mast cell-deficient *Kit^W-sh^* mice and ST2 KO mice. Reconstitution of *Kit^W-sh^* mice with BMMCs from WT mice restores these MWCNT-induced pathological outcomes, whereas that with BMMCs from ST2 KO mice does not. This study therefore demonstrates the crucial role of ST2 in mast cells in promoting MWCNT lung pathology (32). Furthermore, studies using the *Kit^W-sh^* mice demonstrate that mast cells function in increasing the mRNA expression of type 2 cytokines IL-4 and IL-13 by MWCNTs in the lung, indicating the critical role of mast cells in MWCNT-activated type 2 immune response (88). Second, blocking IL-33 signaling by pretreating WT mice with anti-ST2 antibodies markedly reduces the recruitment of eosinophils, but not neutrophils, as well as the levels of IL-5 and CCL11 (Eotaxin), but not IL-6, in the lung exposed to MWCNTs for 24 h. Moreover, the recruitment of eosinophils is impaired in IL-13 KO mice and IL-33 KO mice, but is unaffected in Rag1 KO mice that lack mature B and T cells, compared with that in WT mice (33). Third, the number of eosinophils in BAL is increased by MWCNTs on day 30 post-exposure in WT mice, which is markedly attenuated in IL-33 KO mice (34). Together, these observations strongly support that IL-33−ST2 signaling plays an essential role in stimulating type 2 immune response in MWCNT-exposed lungs.

## IL-4/IL-13−IL-4Rα−STAT6 Signaling

The differentiation and activation of Th2 lymphocytes and M2 macrophages are the hallmark steps in type 2 immune response. Th2 and M2 cells function as the major effector cells to produce a variety of type 2 cytokines and mediators. When type 2 immune response is activated, the early effector cells, such as mast cells, eosinophils, and ILC2s, produce initial IL-4 and IL-13 to stimulate the differentiation of naïve CD4+ T (Th0) cells into Th2 cells. Th2 cells produce large amounts of type 2 cytokines, such as IL-4, IL-5, and IL-13, which serve as the major inducers for the polarization and activation of M2 macrophages. M2 macrophages produce copious amounts of type 2 cytokines and mediators, such as IL-4, IL-10, IL-13, TGF-β1, and PDGF. Through this cascade, type 2 immune response is activated and propagated to exert its biological functions. The cell signaling involved in the activation of Th2 cells and M2 macrophages has been well-characterized, which is featured by the IL-4/IL-13-stimulated, IL-4Rα-mediated activation of STAT6 pathway. In this pathway, IL-4/IL-13 binds to the receptor IL-4Rα to induce phosphorylation of STAT6. Homodimers of phosphorylated STAT6 translocate from cytoplasm to nucleus and transactivate target genes, leading to the expression of type 2 cytokines and mediators. Emerging findings indicate that this signaling cascade is activated during CNT-induced type 2 immune response in the lung, although more comprehensive studies are requisite to address the activities and functions of individual cell types and type 2 cytokines in the future.

A number of studies reveal that type 2 immune response is induced and activated, demonstrated by Th2 cell differentiation, M2 macrophage polarization, and increased production of a variety of type 2 cytokines and mediators, such as IL-4, IL-13, and TGF-β1, in CNT-exposed lungs (10, 14, 15, 52, 82). Meanwhile, significantly increased protein levels of IL-4 in sputum and serum and IL-5 in sputum of the workers exposed to MWCNTs, compared with controls, have been detected (21). Together, these studies provide evidence supporting that type 2 immune response is a critical player in the development of CNT-induced lung inflammation and fibrosis.

It has been visualized that the induction of Th2 cell differentiation and activation occurs in mouse lungs on days 1, 3, 7, and 14 post-exposure to MWCNTs (XNRI MWNT-7). MWCNTs remarkably increase the numbers of IL-4+ CD4+ cells and IL-13+ CD4+ cells, indicating the formation of Th2 cells, as well as the induced expression of IL-4 and IL-13 in Th2 cells. Meanwhile, MWCNTs notably increase the level of phosphorylated STAT6 and the amount of GATA-3, which is upregulated by phosphorylated STAT6 and in turn functions as a transcription factor to transactivate the genes encoding Th2 cytokines, such as IL-4, IL-5, and IL-13, in the lung. The numbers of p-STAT6+ CD4+ cells and GATA-3+ CD4+ cells are evidently elevated in MWCNT-exposed lungs, compared with control lungs. Moreover, a panel of signature downstream target genes of IL-4/IL-13 signaling, including Il4i1, Chia, and Ccl11, are markedly induced by MWCNTs at both mRNA and protein levels in lung tissues. Together, these findings demonstrate the activation of IL-4/IL-13−IL-4Rα−STAT6 signaling in Th2 cells by MWCNTs in the lung (52). The induction of Th2 response by CNTs is supported by a recent study using STAT6 KO mice, in which the level of IL-5 is elevated by MWCNTs (XNRI MWNT-7) in the BAL from WT mice, but not in the BAL from STAT6 KO mice, on day 1 post-exposure (30). Altogether, in response to CNT exposure, Th2 cell differentiation is induced, Th2 hallmark signaling pathway is activated, and Th2-type cytokines are produced, which promote the polarization and activation of M2 macrophages in type 2 immune response.

M2 macrophages promote the function of type 2 immune response through producing type 2 cytokines and mediators. In CNT-exposed lungs, a variety of type 2 mediators, such as TGF-β1, PDGF, IL-10, TIMP1, OPN, and MMP12, are markedly increased, which indicates the activation of M2 macrophages by CNTs (10, 14, 15). Indeed, alongside the increased levels of IL-4 and IL-13 produced by Th2 cells in CNT-exposed lungs, the polarization and activation of M2 macrophages have been demonstrated (82). During the acute response to MWCNTs (XNRI MWNT-7), both M1 and M2 macrophages are induced in mouse lungs. However, M2 macrophages are dominant on days 3 and 7 post-exposure, whereas M1 macrophages mainly exist on days 1 and 3 post-exposure. M2 macrophages are detected with the well-known surface markers, CD206 and CD163. It has been known that in M2 macrophages, but not M1 macrophages, the IL-4/IL-13−IL-4Rα−STAT6 signaling is activated and leads to an increased level of phosphorylated STAT6, and phosphorylated STAT6 directly upregulates the transcription of the genes encoding certain M2 markers and type 2 mediators, such as ARG1, FIZZ1, and YM1 (89, 90). Indeed, phosphorylated STAT6 is markedly induced in MWCNT-exposed lungs, as well as in a subset of macrophages therein, on days 3 and 7 post-exposure, suggesting the activation of IL-4/IL-13−IL-4Rα−STAT6 signaling in M2 macrophages under MWCNT exposure. Concurrently, the levels of ARG1, FIZZ1, and YM1 are dramatically elevated in lung tissues on days 3 and 7 post-exposure to MWCNTs. Furthermore, the induced expression of ARG1 is visualized in a subset of macrophages in MWCNT-exposed lungs on days 3 and 7 post-exposure. Together, these findings disclose the activation of IL-4/IL-13−IL-4Rα−STAT6 signaling in M2 macrophages and the functionalization of M2 macrophages induced by MWCNTs in the lung, leading to the production of type 2 mediators, which underlie the functions of type 2 immune response in MWCNT-induced lung inflammation and fibrosis (82).

In aggregate, accumulative findings have revealed that Th2 lymphocytes and M2 macrophages are induced and activated, and IL-4/IL-13−IL-4Rα−STAT6 signaling is activated to upregulate the expression of type 2 cytokines and mediators in these cells, which together lead to the establishment and propagation of type 2 immune response in CNT-exposed lungs. The activation of type 2 immune response provides the underlying mechanisms for the transition from acute inflammation to Th2-driven type 2 inflammation and chronic inflammation, and for the development of fibrosis that is promoted by various type 2 mediators, such as TGF-β1, TIMP1, and OPN, in CNT-exposed lungs.

## Conclusion and Perspectives

The characteristics of the immune responses induced by CNT exposure in the lung have drawn a great interest to elucidate their underpinning causes, modes of action, and pathological functions. In this regard, the cell signaling pathways and mediators activated during immune responses constitute an essential aspect. A number of crucial signaling pathways activated in immune cells during acute inflammation or type 2 immune response have been revealed in CNT-exposed lungs, which demonstrate a consistency with the knowledge derived from related human lung inflammatory and fibrotic diseases and experimental animal models to a great extent. The activation of these signaling pathways provides the cellular and molecular mechanisms for CNT-induced inflammation and fibrosis, as well as supports the development of CNT-exposed animals as a new model system for dissecting the initiation, transition, progression, and functions of immune responses in lung diseases. The discussion in this article exhibits a mechanistic basis for this emerging, yet promising, research area.

Nevertheless, compared with the numerous pathological observations, the mechanistic understanding of immune responses at the cellular and molecular levels in CNT-exposed lungs is at an early stage and represents a requisite research direction. Regarding the signaling mechanisms activated by CNTs in immune responses in the lung, a number of questions are noticeable to address in future studies. Listed here are a few of them. Which inducing factors, immune cells, cytokines, signaling pathways, and transcription factors, play the determinant roles in the initiation and progression of acute inflammation, type 2 immune response, and chronic inflammation in CNT-exposed lungs? In type 2 immune response, do mast cells, eosinophils, and ILC2s promote Th2 cell differentiation and activation *via* type 2 cytokines, and do Th2 cells control the polarization and activation of M2 macrophages *via* type 2 cytokines? Which type 2 cytokines and mediators function in suppressing acute inflammation? And, which type 2 cytokines and mediators initiate and enhance lung fibrosis? With the facilitation of multiple tools, such as genetically engineered mouse strains, neutralizing antibodies, and specific chemical inhibitors, these questions are expected to address in the coming studies. Meanwhile, cell type-specific analyses of the induced production of pro-inflammatory and pro-fibrotic factors, activated signaling pathways, and pathological functions in CNT-exposed lungs are requisite to further understand the cellular and molecular mechanisms underlying CNT-induced lung inflammation and fibrosis. An intensive research effort and a bloom of new achievements in this research area are anticipated to appear in the coming years, which might lead to new findings to enhance the understanding of human lung diseases involving inflammation and fibrosis.

## Ethics Statement

The animal study was reviewed and approved by the CDC-Morgantown Institutional Animal Care and Use Committee.

## Author Contributions

The author confirms being the sole contributor of this work and has approved it for publication.

## Funding

This work was funded to JD by the Health Effects Laboratory Division and the Nanotechnology Research Center at National Institute for Occupational Safety and Health, Centers for Disease Control and Prevention, USA (No. 9390DU8).

## Disclaimer

The findings and conclusions in this report are those of the author and do not necessarily represent the official position of the National Institute for Occupational Safety and Health, Centers for Disease Control and Prevention.

## Conflict of Interest

The author declares that the research was conducted in the absence of any commercial or financial relationships that could be construed as a potential conflict of interest.

## Abbreviations

4-HNE, 4-hydroxy-2-nonenal; 8-OHdG, 8-hydroxy-2′-deoxyguanosine; α-SMA, α-smooth muscle actin; AP-1, activator protein 1; ARG1, arginase 1; ASC, apoptosis-associated speck-like protein containing a caspase activation and recruitment domain; BAL, bronchoalveolar lavage; BMMC, bone marrow-derived mast cell; CCL, chemokine (C-C motif) ligand; CD163, hemoglobin scavenger receptor; CD206, mannose receptor C-type 1 or MRC1; Chia, chitinase, acidic, or AMCase; CNT, carbon nanotube; COX-2, cyclooxygenase-2; CXCL, chemokine (C-X-C motif) ligand; ECM, extracellular matrix; ELISA, enzyme-linked immunosorbent assay; ERK, extracellular signal-regulated kinase; FGF-BASIC, basic fibroblast growth factor; FIZZ1, found in inflammatory zone 1, resistin-like molecule α or RELMα, or resistin-like-α or RETNLα; γH2AX, phospho-Histone H2A.X (Ser139); GATA-3, GATA-binding protein 3; GSH, glutathione; HMGB1, high mobility group box 1; ICAM-1, intercellular adhesion molecule 1; IL, interleukin; IL-1R, IL-1 receptor; IL-1RAcP, IL-1 receptor accessory protein; Il4i1, interleukin 4 induced 1, or Fig1; IL-4Rα, IL-4 receptor α; IL-6R, IL-6 receptor; ILC2, group 2 innate lymphoid cell; JNK, c-Jun N-terminal kinase; KO, knockout; LPS, lipopolysaccharides; M1, traditionally activated macrophage; M2, alternatively activated macrophage; MAPK, mitogen-activated protein kinase; MCP-1, monocyte chemotactic protein 1; MKK, mitogen-activated protein kinase kinase; MMP12, matrix metalloproteinase 12; MWCNT, multi-walled carbon nanotube; NF-κB, nuclear factor-κB; NLR, nucleotide-binding oligomerization domain-like receptor; NLRP3, nucleotide-binding oligomerization domain-like receptor: pyrin domain-containing 3; Nrf2, nuclear factor erythroid 2-related factor 2; OPN, osteopontin or secreted phosphoprotein 1 or SPP1; PDGF, platelet-derived growth factor; Rag1, recombination activating gene 1; ROS, reactive oxygen species; sIL-1RII, soluble IL-1 receptor II; ST2, suppression of tumorigenicity 2, receptor for IL-33 or IL-33R, IL-1 receptor-like 1 or IL1RL1, or IL-1 receptor 4 or IL-1R4; STAT6, signal transducer and activator of transcription 6; SWCNT, single-walled carbon nanotube; Syk, spleen tyrosine kinase; Th, T helper; TGF-β1, transforming growth factor-β1; TIMP1, tissue inhibitor of metalloproteinase 1; TNF-α, tumor necrosis factor-α; TNFR, TNF receptor; TSLP, thymic stromal lymphopoietin; WT, wild-type; YM1, chitinase 3-like 3 or CHI3L3, or eosinophil chemotactic factor-lymphocyte or ECF-L.
